# COVID-19 vaccination and carditis in children and adolescents: a systematic review and meta-analysis

**DOI:** 10.1007/s00392-022-02070-7

**Published:** 2022-07-30

**Authors:** Oscar Hou In Chou, Jonathan Mui, Cheuk To Chung, Danny Radford, Simon Ranjithkumar, Endurance Evbayekha, Ronald Nam, Levent Pay, Danish Iltaf Satti, Sebastian Garcia-Zamora, George Bazoukis, Göksel Çinier, Sharen Lee, Vassilios S. Vassiliou, Tong Liu, Gary Tse, Ian Chi Kei Wong, the Cardiovascular Analytics Group, the International Health Informatics Study Network, Oscar Hou In Chou, Tong Liu, Gary Tse

**Affiliations:** 1 Epidemiology Research Unit, Cardiovascular Analytics Group, China-UK Collaboration, Hong Kong, China; 2 Kent and Medway Medical School, Canterbury Christ Church University, Canterbury, UK; 3 Faculty of Health and Life Sciences, Aston University Medical School, Birmingham, B4 7ET UK; 4 Department of Cardiology, Dr Siyami Ersek Thoracic and Cardiovascular Surgery Training and Research Hospital, 34668 Istanbul, Turkey; 5 Cardiac Intensive Care Unit, Department of Cardiology, Delta Clinic, Rosario, Santa Fe Argentina; 6 Department of Cardiology, Medical School, University of Nicosia, 2408 Nicosia, Cyprus; 7 Department of Medicine, Bob Champion Research and Education, Norwich Medical School, University of East Anglia and Norfolk and Norwich University Hospital, Rosalind Franklin Rd, Norwich, NR4 7UQ UK; 8 Tianjin Key Laboratory of Ionic-Molecular Function of Cardiovascular Disease, Department of Cardiology, Tianjin Institute of Cardiology, Second Hospital of Tianjin Medical University, Tianjin, China; 9 Research Department of Practice and Policy, School of Pharmacy, University College London, London, UK; 10 Kent and Medway Medical School, University of Kent, Canterbury, United Kingdom

**Keywords:** COVID-19, Vaccine, Myocarditis, Pericarditis, Carditis

## Abstract

**Background:**

Coronavirus Disease-2019 (COVID-19) vaccination has been associated with the development of carditis, especially in children and adolescent males. However, the rates of these events in the global setting have not been explored in a systematic manner. The aim of this systematic review and meta-analysis is to investigate the rates of carditis in children and adolescents receiving COVID-19 vaccines.

**Methods:**

PubMed, Embase and several Latin American databases were searched for studies. The number of events, and where available, at-risk populations were extracted. Rate ratios were calculated and expressed as a rate per million doses received. Subgroup analysis based on the dose administered was performed. Subjects ≤ 19 years old who developed pericarditis or myocarditis following COVID-19 vaccination were included.

**Results:**

A total of 369 entries were retrieved. After screening, 39 articles were included. Our meta-analysis found that 343 patients developed carditis after the administration of 12,602,625 COVID-19 vaccination doses (pooled rate per million: 37.76; 95% confidence interval [CI] 23.57, 59.19). The rate of carditis was higher amongst male patients (pooled rate ratio: 5.04; 95% CI 1.40, 18.19) and after the second vaccination dose (pooled rate ratio: 5.60; 95% CI 1.97, 15.89). In 301 cases of carditis (281 male; mean age: 15.90 (standard deviation [SD] 1.52) years old) reported amongst the case series/reports, 261 patients were reported to have received treatment. 97.34% of the patients presented with chest pain. The common findings include ST elevation and T wave abnormalities on electrocardiography. Oedema and late gadolinium enhancement in the myocardium were frequently observed in cardiac magnetic resonance imaging (CMR). The mean length of hospital stay was 3.91 days (SD 1.75). In 298 out of 299 patients (99.67%) the carditis resolved with or without treatment.

**Conclusions:**

Carditis is a rare complication after COVID-19 vaccination across the globe, but the vast majority of episodes are self-limiting with rapid resolution of symptoms within days.

**Graphical abstract:**

Central illustration. Balancing the benefits of vaccines on COVID-19-caused carditis and post-vaccination carditis.

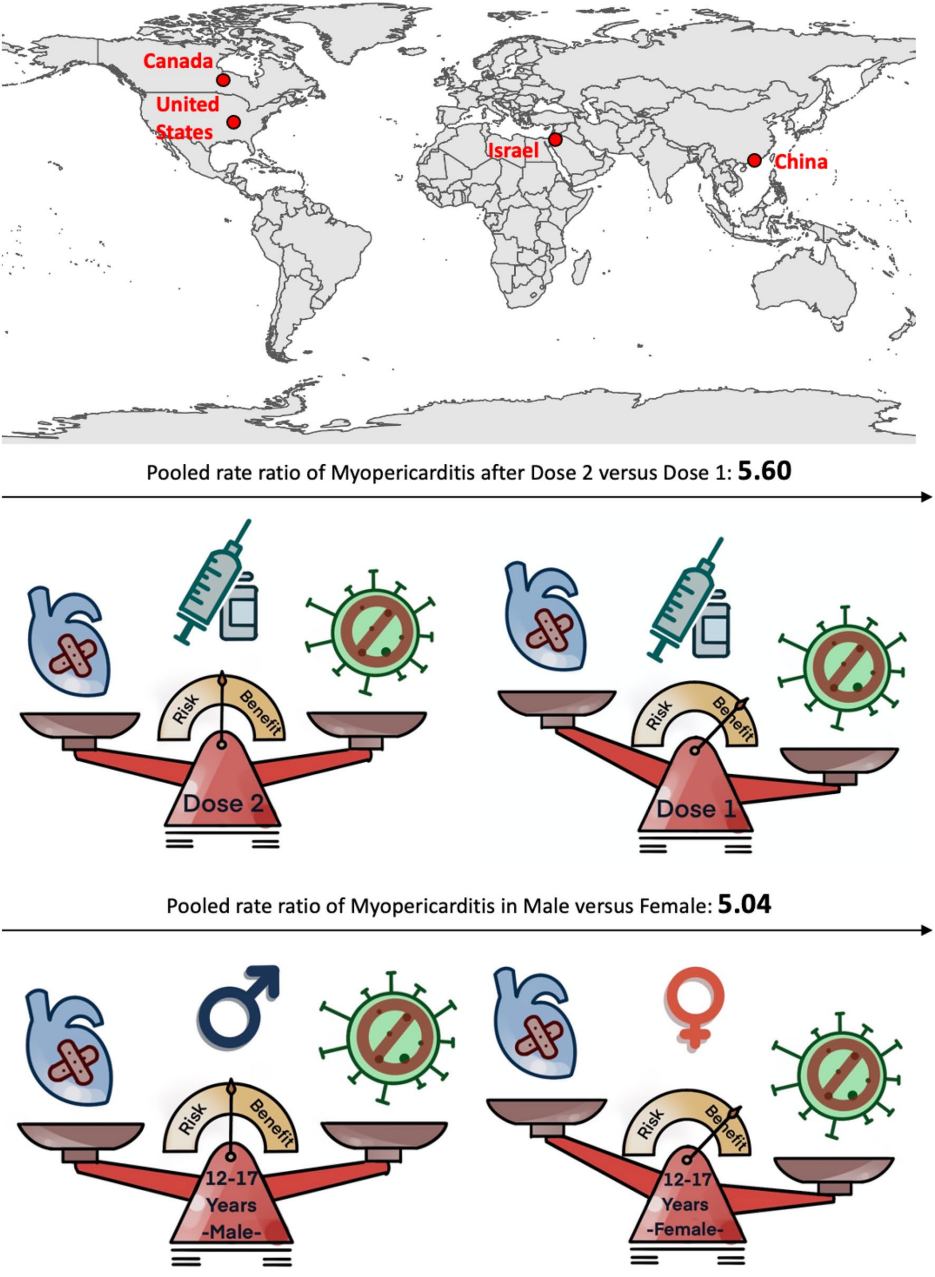

**Supplementary Information:**

The online version contains supplementary material available at 10.1007/s00392-022-02070-7.

## Introduction

Vaccination for the Coronavirus Disease 2019 (COVID-19) has provided significant protection against the development of severe complications and mortality, reducing the burden on healthcare systems globally. The development and rollout of vaccination have occurred at an unprecedented rate internationally. As of March 6th, 2022, more than 10 million vaccine doses have been administered across the world [1]. Previous studies have shown that the approved COVID-19 vaccines are effective in reducing COVID-19 infection, hospitalisation, and mortality rates [2–4]. A recent systematic review evaluating 11 COVID-19 vaccines showed that the 28 day seroconversion rate was over 80% and most adverse reactions were local, mild, and self-limiting within 24 h of vaccination [5].

However, while the vaccines showed favourable safety profiles in early clinical trials and post-marketing reports [4, 6–8], rare adverse events have subsequently been reported [9, 10]. Of these, carditis is the acute inflammation of the heart and can involve the myocardium and/or the pericardium. Vaccine-related carditis is rare and has previously been reported with live-attenuated vaccines such as smallpox and influenza vaccines [2, 11, 12]. Since the approval of COVID-19 vaccines for emergency use, case reports or series on post-vaccine myopericarditis have been published [13, 14]. This has been noted and investigated by safety agencies internationally, including the Centre for Disease Control and Prevention (CDC) in the United States and the Pharmacovigilance Risk Assessment Committee (PRAC) in Europe [15, 16]. Analysis across age-groups and vaccine type suggests the risk of carditis is primarily elevated among young male patients [17, 18] following mRNA vaccination such as the Pfizer-BioNTech (BNT 162b2) and Moderna (mRNA-1273) vaccines [19, 20].

Our team has recently examined the overall rates of myopericarditis in Asia and compared them to those reported in other countries [21, 22]. We found that the rate of vaccine-related myopericarditis among adults is similar to the background rate but much lower than COVID-19 infection, while the rate of myopericarditis among 12–15 years old patients was higher [21, 23]. However, there has been no systematic evaluation of the paediatric, adolescent or young adult subgroups, which are thought to be affected to greater extents than older adults. Therefore, the aim of this systematic review and meta-analysis is to investigate the rates of carditis in paediatric subjects receiving COVID-19 vaccines.

## Methods

### Search strategy, inclusion and exclusion criteria

The systematic review and meta-analysis were performed according to the Preferred Reporting Items for Systematic Reviews and Meta-Analyses (PRISMA) statement. It was registered with the International Prospective Register of Systematic Reviews (PROSPERO) (Registration Number: CRD42022315126). PubMed, Embase and the Latin American databases LILACS, BRISA/RedTESA, IBECS, LIPECS, Sec. Est. Saúde SP, Scielo were searched from inception to March 6th, 2022 for published studies describing subjects ≤ 19 years old who developed pericarditis or myocarditis following COVID-19 vaccination. The search terms for PubMed were: ((pericarditi s [Title/Abstract]) OR (myocarditis [Title/Abstract]) OR (cardiac [Title/Abstract])) AND (covid19 [Title/Abstract]) AND (vaccin*[Title/Abstract]) AND ((child*[Title/Abstract]) OR (adolescent*[Title/Abstract]) OR (paediatric [Title/Abstract]) OR (young [Title/Abstract])), and those for Embase were: (pericarditis:ab,ti OR myocarditis:ab,ti OR cardiac:ab,ti) AND (covid-19’:ab,ti AND vaccin*:ab,ti) AND (children:ab,ti OR adolescent:ab,ti OR paediatric:ab,ti OR young:ab,ti). The Latin American databases LILACS, BRISA/RedTESA, IBECS, LIPECS, Sec. Est. Saúde SP, Scielo were also searched using the following search terms: 1. (miocarditis) AND (vacuna) AND (COVID) OR (coronavirus); 2. (pericarditis) AND (vacuna) AND (COVID) AND (coronavirus) (Supplementary Table 1).

### Screening, data extraction and quality assessment

Search results for PubMed, Embase and the Latin American databases were independently screened by team members. Each entry was screened by two members separately and assessed for compliance with the inclusion criteria. The exclusion criteria during data screening are included in Supplementary Table 2. Any disagreements would be brought to the attention of an independent reviewer not involved in the initial screening (GT). Data from the different studies were entered in Microsoft Excel. The initial plan was to search the largest passive vaccine surveillance systems, EudraVigilance, Vaccine Adverse Event Reporting System (VAERS) and VigiBase databases for adverse events. However, both VAERS and VigiBase were analysed by the included studies, and therefore these databases were not searched ultimately to avoid the duplication of data. In this meta-analysis, the extracted data elements consisted of: (1) last name of the first author and year of publication; (2) study type, (3) country of study; (4) number of events and number of at-risk individuals, (5) follow-up duration of the study, (6) sex and age, (7) vaccine type, (8) number of doses received, (9) delay between vaccination and onset of carditis, (10) definition of carditis, (11) treatment received, (12) number of cases that recovered with or without treatment, (13) length of hospital stay by the myocarditis/pericarditis cases. Subgroup analysis based on the type of vaccine was performed. Quality evaluation of case reports/case series was assessed using the case-report (CARE) 13-item guideline [24, 25]. Case–control and cohort studies were evaluated using the Newcastle–Ottawa Quality Assessment Scale [26].

### Statistical analysis

Summary statistics were used to summarise the number of events, age, sex, vaccine types and outcomes for all cases described by all studies. From population-based studies, the number of events and where available, at-risk populations were extracted. Rate ratios were stratified according to dose and gender and expressed as a rate per million doses received. The confidence interval of the rates was calculated using the Poisson Exact Method. The heterogeneity across the studies was determined using Cochran’s *Q* value and the *I*^2^ statistics. The fixed-effect model was used to conduct the meta-analysis. However, the random-effects model was used to calculate the pooled rate across the studies when the heterogeneity was significant (*I*^2^ > 50%). The rate ratios were calculated to compare the rate of carditis after COVID-19 vaccination and infection. The analysis was performed using RStudio (version 1.4.1103).

## Results

**Fig. 1:**
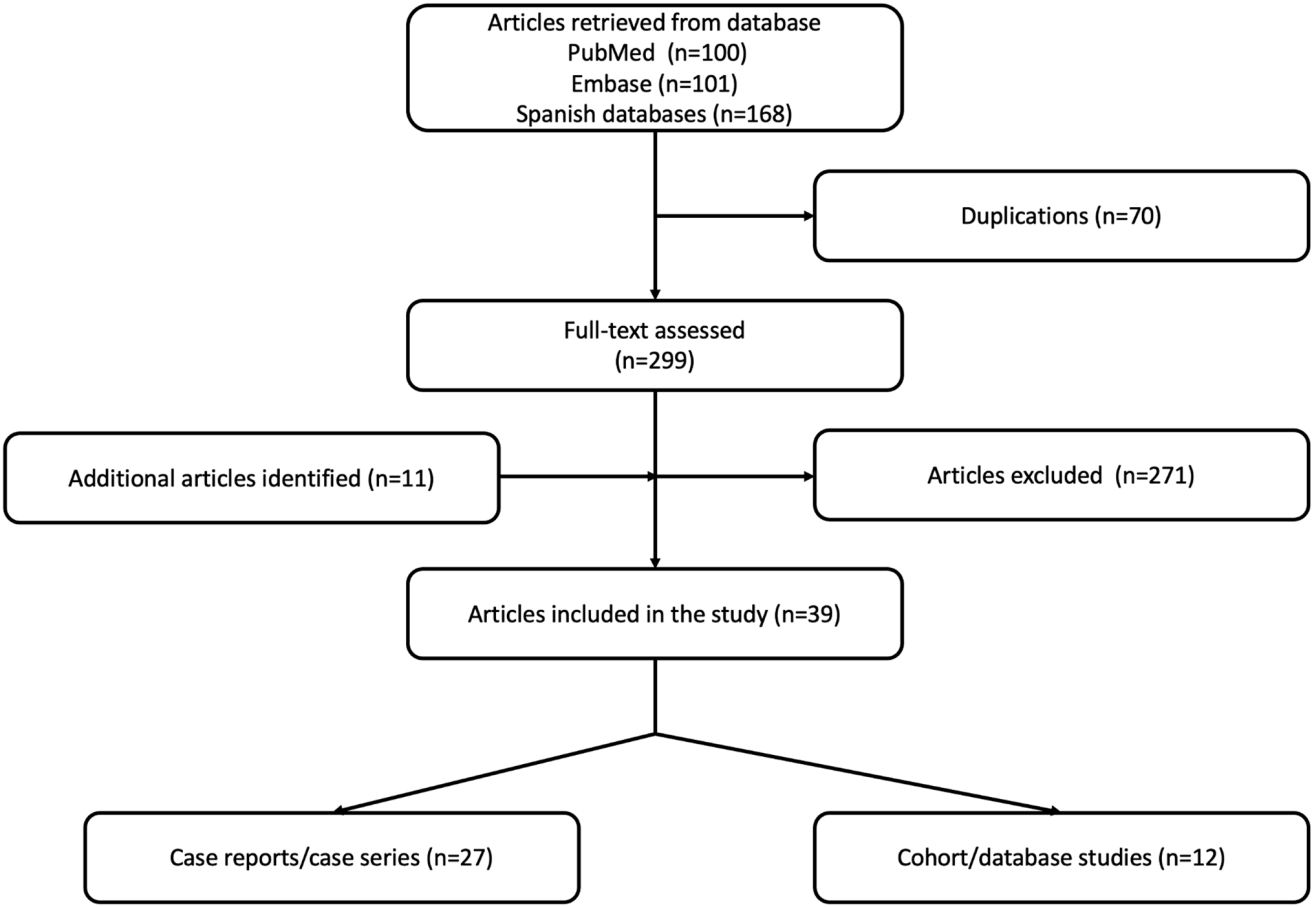
The flowchart of the database search and study selection

The Quality of Reporting of Meta-analyses standards (QUORUM) diagram detailing the above search terms with the inclusion and exclusion criteria is depicted in Fig. 1. A total of 101 from Embase, 100 entries from Pubmed, and 168 entries from the Latin American databases were retrieved. After screening, 39 articles were included after including the additional articles identified.

### Worldwide rates of carditis

Twelve population-based studies were included in the study (Table 1). In total, 12,602,625 doses, including 8,050,898 first doses and 4,551,727 s doses, were administered after excluding overlapping cohorts. Overlapping cohorts were identified from analysing the methodology of the studies and defined as patient cohorts in the same country and/or hospitals within a similar time period. All of the subjects (49.95% male) were vaccinated with BNT162b2. Five population-based studies involving individuals receiving COVID-19 vaccines from the United States, Canada, Israel and Hong Kong, China were selected after excluding the overlapping cohorts or cohorts without providing information on the doses administered.

**Table 1:** Cohort/case–non case/case–control studies included (*n* = 12)

Author (last name)	Year published	Study location (country)	Study design
Bozkurt	2021	United States (VAERS)	Vaccine reporting system
Chouchana	2022	Global (Vigibase)	Case–non-case study
Chua*	2021	Hong Kong, China	Cohort study
Foltran	2021	Global (Vigibase)	Vaccine reporting system
Hause	2021	United States	VAER report
Krug*	2022	United States	Vaccine reporting system
Li	2022	China	Case–control study
Truong*	2021	United States (VAERS)	Vaccine reporting system
Li *	2021	Hong Kong, China	Cohort study
Buchan*	2021	Canada	Cohort study
Mevorache*	2021	Israel	Cohort study
Nygaard	2022	Denmark	Cohort study and case series

A total of 12 studies were included. Only the studies that provide the total number of doses were included in calculating the rate of myopericarditis per million dose of vaccine (marked with asterisks). Only the study with the largest sample size was selected amongst studies with overlapping data

**Fig. 2:**
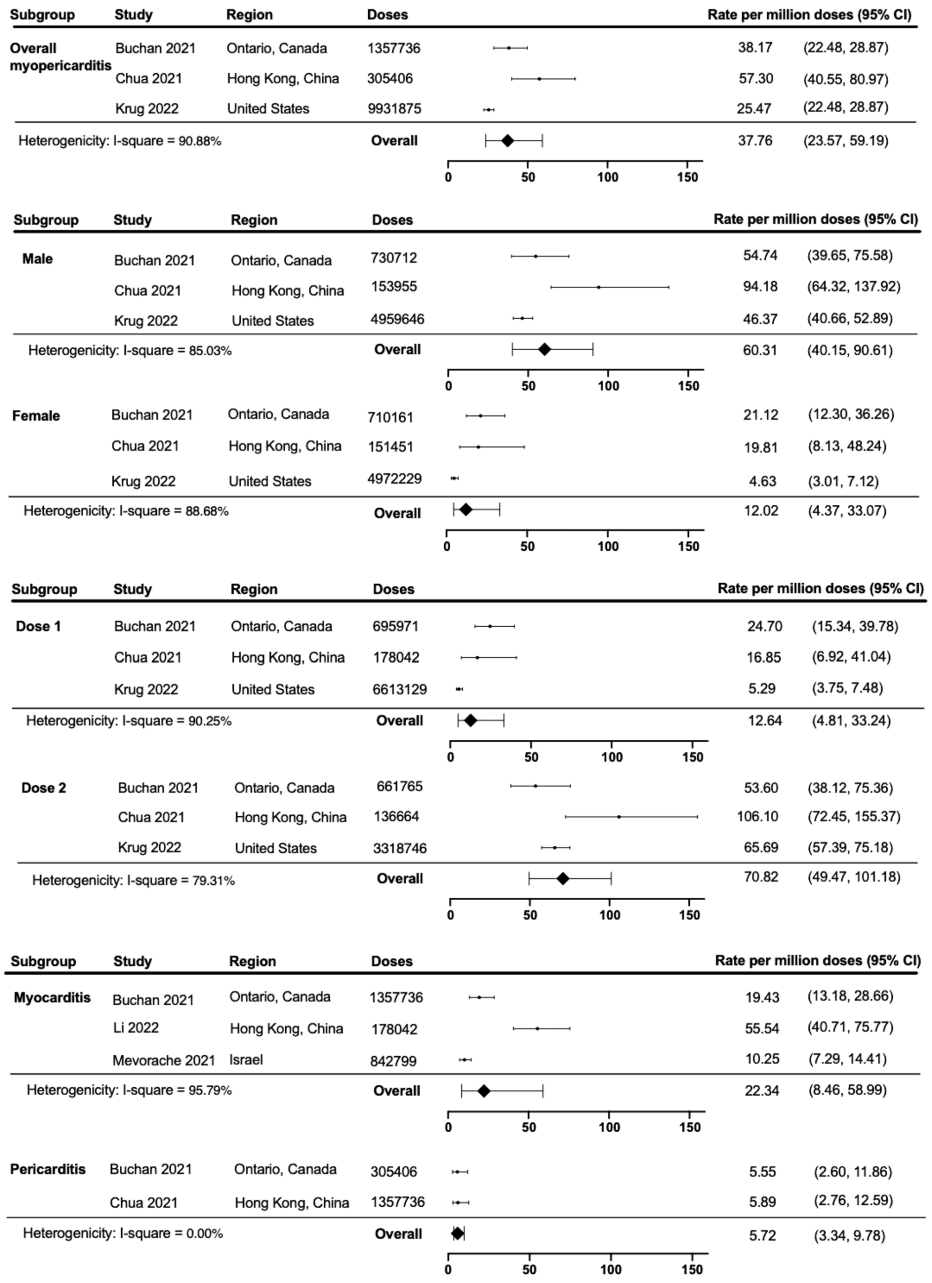
The rate of carditis after COVID-19 vaccination with subgroup analysis. The overall rate of carditis amongst the paediatrics patients were calculated using the Poisson exact method. The rate of carditis were also categorised by sex, dosage and myocarditis/pericarditis

Of the pooled studies reporting on carditis, 343 patients developed carditis, corresponding to a rate per million doses per 7 days of 37.76 (95% confidence interval [CI] 23.57, 59.19) (Fig. 2). Further subgroup analyses by sex showed that the rate of carditis was higher among males (rate per million: 60.31; 95% CI 40.15, 90.61) than females (rate per million: 12.02; 95% CI 4.37, 33.07) with a rate ratio of 5.04 (1.40, 18.19) (Table 2). The rate of carditis was significantly higher among the second dose (rate per million: 70.82; 95% CI 49.47, 101.18) than the first dose (rate per million: 12.64; 95% CI 4.81, 33.24) with a rate ratio of 5.60 (95% CI 1.97, 15.89). From the studies providing myocarditis-only data, the rate per million doses of myocarditis was 22.34 (95% CI 8.46, 58.99). The rate per million doses of pericarditis was 5.72 (95% CI 3.34, 9.87).

**Table 2:** The pooled rate ratio of carditis after COVID-19 vaccination between male versus female and dose 2 versus dose

Comparison	Pooled rate ratio (95% confidence interval)
Male vs female	5.04 (1.40, 18.19)
Dose 2 vs dose 1	5.60 (1.97, 15.89)

The rate of carditis per 7 days in the background and after COVID-19 infection were carefully selected by matching the location of the selected studies. Overall, among paediatric patients, the rates of carditis after the COVID-19 vaccination were not significantly different from the COVID-19 infection (Table 3). The rate of carditis after the first dose of vaccination was lower than the COVID-19 infection. Meanwhile, the rate of carditis after the second dose was higher than the background and not different from the COVID-19 infection. For male patients, the rate of carditis was significantly higher than the background, similar to that after COVID-19 infection. For females, the rate of carditis was much lower than that after COVID-19 infection.

**Table 3:** Comparing the rate of carditis after COVID-19 vaccination to the background rate and after COVID-19 infection by countries

		Vaccination			Background			COVID-19 infection		
Study	Location	Age group	Rate per million	Rate ratio	Age group	Rate per million	Rate ratio	Age group	Rate per million	Rate ratio
Overall										
Buchan 2021 [55]	Ontario, Canada	12–17	38.17 (28.76, 38.17)	1	16–19	2.570 (2.071, 3.145) [56]	0.0673 (0.0193, 0.2351)	–	–	–
Chua 2021[42]	Hong Kong, China	12–17	57.30 (39.91, 79.69)	1	12–17	2.3 (0.275, 16.08) [42]	0.0401 (0.0021, 0.7359)	–	–	–
Oster 2022^b^[14]	United States	12–17	53.89 (49.27, 58.95)	1	15–18^a^	0.2877 (0.28, 0.2953) [57]	0.0053 (0.0041, 0.0070)	12–17	44.34 (22.56, 93.66) [58]	0.8228 (0.2770, 2.444)
Mevorach 2021^b^ [59]	Israel	16–19	10.25 (7.176, 14.19)	1	–	–	–	16–19	14.15 (3.855, 38.23) [59]	1.381 (0.4915, 3.880)
Dose 1										
Buchan 2021 [55]	Ontario, Canada	12–17	24.70 (14.87, 38.57)	1	16–19	2.570 (2.071, 3.145) [56]	0.1041 (0.0283, 0.3828)	–	–	–
Chua 2021[42]	Hong Kong, China	12–17	16.85 (6.183, 36.76)	1	12–17	2.3 (0.275, 16.08) [42]	0.1365 (0.0068, 2.737)	–	–	–
Oster 2022^b^[14]	United States	12–17	5.85 (4.41, 7.76)	1	15–18^a^	0.2877 (0.28, 0.2953) [57]	0.0492 (0.0219, 0.1107)	12–17	44.34 (22.56, 93.66) [58]	7.579 (2.003, 28.68)
Dose 2										
Buchan 2021 [55]	Ontario, Canada	12–17	53.60 (37.54, 74.20)	1	16–19	2.570 (2.071, 3.145) [56]	0.0479 (0.0135, 0.1700)	–	–	–
Chua 2021[42]	Hong Kong, China	12–17	106.1 (71.06, 153.4)	1	12–17	2.3 (0.275, 16.08) [42]	0.0217 (0.0012, 0.3990)	–	–	–
Oster 2022^b^[14]	United States	12–17	69.06 (62.83, 75.90)	1	15–18^a^	0.2877 (0.28, 0.2953) [57]	0.0042 (0.0033, 0.0053)	12–17	44.34 (22.56, 93.66) [58]	0.6421 (0.2177, 1.893)
Male										
Buchan 2021[55]	Ontario, Canada	12–17	54.74 (39.11, 74.54)	1	16–19	2.570 (2.071, 3.145) [56]	0.0470 (0.0133, 0.1657)	–	–	–
Chua 2021[42]	Hong Kong, China	12–17	94.18 (63.07, 135.3)	1	12–17	2.3 (0.275, 16.08) [42]	(0.0244 (0.0013, 0.4494)	–	–	–
Oster 2022^b^[14]	United States	12–17	66.54 (60.57, 73.11)	1	15–18^a^	0.2877 (0.28, 0.2953) [57]	0.0043 (0.0034, 0.0055)	12–17	44.34 (22.56, 93.66) [58]	0.6663 (0.2257, 1.967)
Female										
Buchan 2021[55]	Ontario, Canada	12–17	21.12 (11.82, 34.84)	1	16–19	2.570 (2.071, 3.145) [56]	0.1216 (0.0150, 0.2797)	–	–	–
Chua 2021[42]	Hong Kong, China	12–17	19.81 (7.269, 43.11)	1	12–17	2.3 (0.275, 16.08) [42]	0.1151 (0.0058, 2.328)	–	–	–
Oster 2022^b^[14]	United States	12–17	6.71 (4.99, 9.02)	1	15–18^a^	0.2877 (0.28, 0.2953) [57]	0.0429 (0.0201, 0.0915)	12–17	44.34 (22.56, 93.66) [58]	6.608 (1.803, 24.21)

^a^The background rate of the United States contains myocarditis only

^b^The rate in Mevorach et al. and Oster et al. contains myocarditis only

### Clinical characteristics

**Fig. 3:**
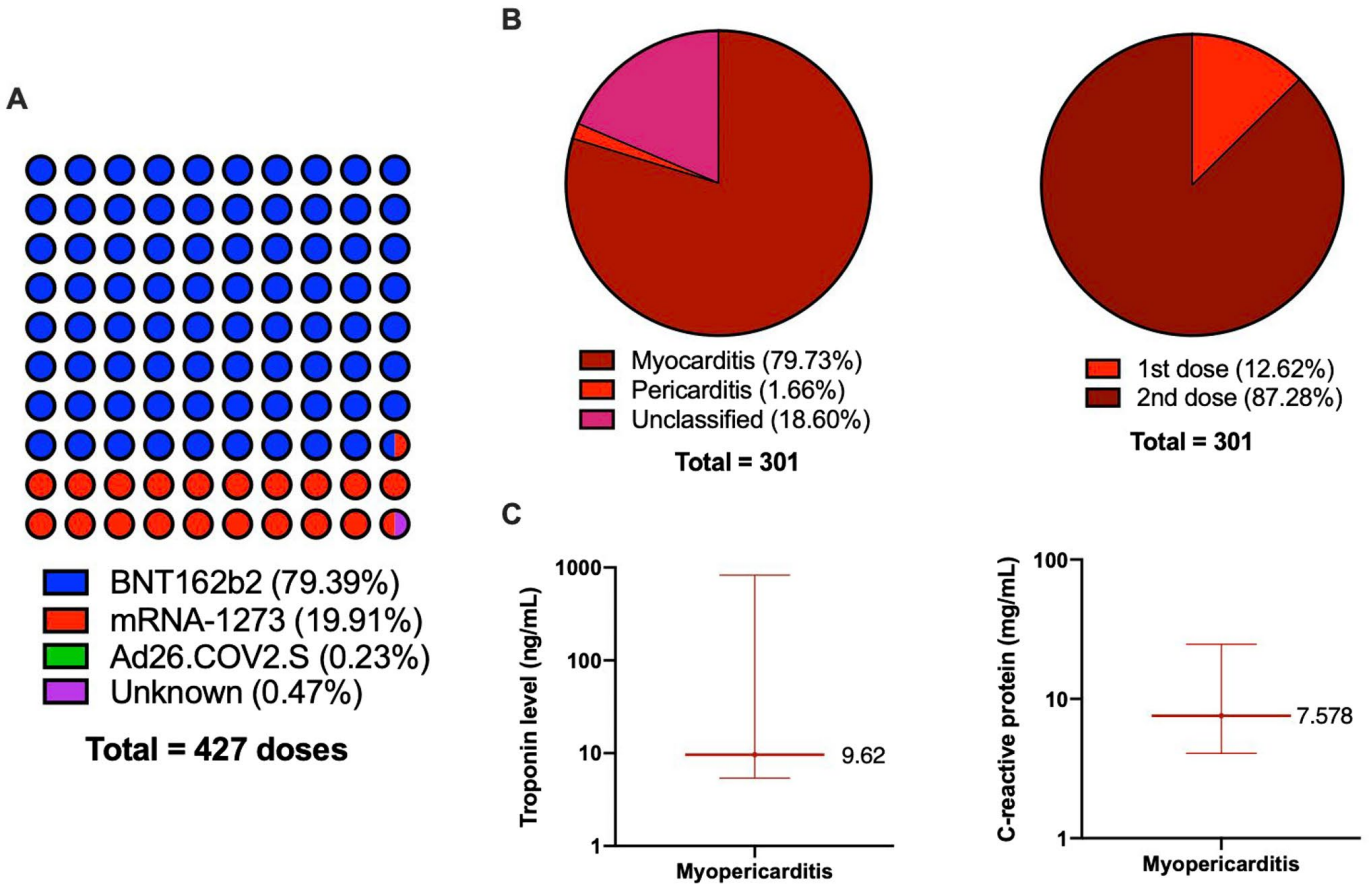
The characteristics of the carditis patients after COVID-19 vaccination. **A** The proportion of different types of a vaccine in the case reports/series. **B** The diagnosis and timing of carditis in the case reports/series. **C** The median and interquartile range of Troponin and C-reactive protein in the case reports/series

A total of 301 patients who developed carditis were included (Table 4). 281 cases were male with a mean age of 15.90 (SD 1.52) (Table 5). Of all doses vaccinated, 79.39% were BNT162b2 (Pfizer) vaccine, 19.91% were Moderna (mRNA-1273) vaccine, and < 0.23% were Ad26.COV2.S (Johnson & Johnson’s Janssen) vaccine (Fig. 3A). Some 240 (79.73%) had myocarditis, 5 (1.66%) had pericarditis and 56 (18.60%) had unclassified carditis. A total of 38 (12.62%) events occurred after the first dose and 263 (87.38%) events occurred after the second dose (Fig. 3B). Only 3 patients reported a previous history of cardiac diseases.

**Table 4:** Case studies/series included (*n* = 27)

Author (last name)	Year published	Study location (Country)	Study design	Total number of myopericarditis
Ambati	2021	United States	Case series	2
Azir	2021	United States	Case report	1
Buchhorn	2021	Germany	Case report	1
Chelala	2022	United States	Case series	5
Das	2021	United States	Case series	25
Di	2022	Italy	Case series	1
Dionne	2021	United States	Case series	15
Giray	2022	Turkey	Case report	1
Manfredi	2022	Italy	Case series	6
Marshall	2021	United States	Case series	7
McLean	2021	United States	Case study	1
Minocha	2021	United States	Case study	1
Poussaint	2021	United States	Case report	1
Snapiri	2021	Israel	Case series	7
Tano	2021	United States	Case series	8
Truong	2022	Canada, United States	Case report	140
Türe	2022	Turkey	Case report	1
Visclosky	2021	United States	Case report	1
Jain	2021	United States	Case series	31
Starekova	2021	United States	Case series	2
Abu Mouch	2021	Israel	Case series	2
Fleming-nouri	2021	United States	Case series	3
Park	2021	United States	Case series	2
Shaw	2021	United States	Case series	2
Patel	2021	United States	Case series	9
Schauer	2021	United States	Case series	13
Nygaard	2022	Denmark	Cohort study and case series	13

**Table 5:** The clinical characteristics of the patients ≤ 19 years old suffering from carditis after COVID-19 vaccination

Patient characteristics (*n* = 301)	Mean/number	Standard deivation/%
Patient demographics		
Age (year)	15.90	1.52
Male	281	93.36%
Clinical diagnosis		
Total carditis	301	100.00%
Total myocarditis	240	79.73%
Total pericarditis	5	1.66%
Unclassified	56	18.60%
Subgroup		
Carditis after first dose	38	12.62%
Carditis after second dose	263	87.38%
Pericarditis after first dose	3	60.00%
Pericarditis after second dose	2	40.00%
Myocarditis after first dose	30	12.50%
Myocarditis after second dose	210	87.50%
Proportion of vaccine types		
BNT162b2	339	79.39%
mRNA-1273	85	19.91%
Ad26.COV2.S	1	0.23%
Unknown	2	0.47%
Admission		
Length of stay (days)	3.905	1.75
Intensive care unit	53	20.31
Resolution	298	99.00%
Clinical manifestation		
Chest pain	293	97.34%
Fever	113	37.54%
Shortness of breath	64	21.26%
Myalgia	51	16.94%
Headache	47	15.61%
Nausea and vomiting	31	10.30%
Palpitations	8	2.66%
Laboratory results		
Troponin (ng/mL)	924.32	2017.01
Median (Q1 to Q3)	9.62 (5.40–828.09)	–
Min; max	0.03; 7368.45	–
C-reactive protein level (mg/mL)	25.89	44.31
Median (Q1–Q3)	7.58 (4.06–24.70)	
Min; max	0.57; 174	
Treatment		
Treatment received	261	86.71%
NSAIDs	226	86.59%
Steroids	50	19.16%
Intravenous immunoglobulins	57	21.84%
Angiotensin-converting enzyme (ACE) inhibitors	2	0.77%
Colchicine	24	9.20%

The most common presenting symptoms were: chest pain (97.34%), fever (37.54%), dyspnoea (21.26%), myalgia (16.94%) and headache (15.61%). Laboratory findings included elevated troponin (median: 9.62; interquartile range [IQR] 4.40, 828.09) and C-reactive protein levels (median: 7.58; IQR 4.06, 24.70) (Fig. 3C). The most common findings in the electrocardiogram included ST elevation and T wave abnormalities. Other findings such as ST depression, non-specific ST changes, interventricular conduction, PR depression, atrial tachycardia and left axis deviation were also reported. Four patients reported to have sinus tachycardia. Common CMR findings included oedema and late gadolinium enhancement in the myocardium. The echocardiogram abnormalities included decreased left ventricular ejection fraction and pericardial effusion.

Overall, 261 (86.71%) patients were reported to have received treatment. 53 (20.31%) patients required intensive care unit admission and the mean length of stay in the hospital was 3.91 days (SD 1.75) (Table 3). Among those receiving treatment, 226 (86.59%) received nonsteroidal anti-inflammatory agents (NSAID) and 50 (19.16%) received steroid treatment. Intravenous immunoglobulins were prescribed in 57 (21.84%) patients. Angiotensin-converting enzyme (ACE) inhibitors and colchicine were also used in 2 (0.77%) and 24 (9.20%) patients, respectively. Out of the 299 cases with longitudinal description, 298 (99.67%) patients had resolution of the myocarditis and/or pericarditis with or without treatment and none of the patients died as a result of the myocarditis and/or pericarditis.

## Discussion

To the best of our knowledge, this is the first systematic review and meta-analysis of COVID-19 vaccine-related carditis in the paediatric population. We found that carditis is a rare complication following COVID-19 vaccination, with 343 cases of carditis observed over an at-risk population of 12,602,625 doses (0.0027%). The majority of cases occurred after receiving the second dose of the vaccine. In addition, most of the cases were self-limiting and resolved within seven days after medical treatment. Vaccinating with the first dose of COVID-19 was associated with a lower rate of carditis than those infected with COVID-19. Furthermore, the rate was significantly lower among females compared to being infected with COVID-19.

### Comparison with previous studies

The landmark trials of COVID-19 vaccinations demonstrated favourable safety profiles with complications being rarely reported, with no cases of post-vaccine myocarditis or pericarditis being observed [27, 28]. This could have been due to the small number of patients enrolled in the clinical trials, as well as the apparent rarity of this complication [29]. However, some concerns were raised following the reports by the CDC and VAERS of rare cases of myocarditis and pericarditis being associated with COVID-19 mRNA vaccinations, specifically the Pfizer-BioNTech mRNA vaccine (BNT162b2) and the Moderna mRNA vaccine (mRNA-1273) [14, 30–35]. Since the COVID-19 vaccine was only approved for adults at the beginning, the majority of the reported side effects were studied in adults, with little attention paid to children and adolescents. Nonetheless, it was reported that the risk of myocarditis and pericarditis following COVID-19 vaccination was the highest among males and young adults [36–38]. Our review further extends these observations, by demonstrating an increased risk among those aged 12–17 years and of the male sex.

Our findings suggest that the risk of post-vaccine carditis is highest among males aged 12–17 years old [14]. This is likely related to the age and sex distribution of non-vaccine-associated myocarditis in the general population, demonstrating a bimodal distribution of incidence peaking at infancy and adolescence more common in males [39–41]. We also noted that the risk of post-vaccine carditis is higher in Asian populations than in Western populations, with Chua et al*.* and Li et al*.* reporting notably higher rates of post-vaccine carditis than in other studies [42, 43]. This is possibly due to the increased monitoring and reporting of cases in such areas, leading to increased detection of mild cases of carditis [44]. However, this may also relate to the general epidemiology of myocarditis, with the Global Burden of Disease study reporting the highest incidence of myocarditis in 2017 in East Asia and South Asia [23].

### Clinical implications and the future

Although vaccine-related carditis has attracted widespread attention in the media, it is a rare complication. Its severity is usually mild to moderate with an early clinical diagnosis following (CMR) [45]. It was previously proposed that post-vaccination carditis can be due to an immune-mediated adverse response induced by the vaccination [46]. Alternatively, it was also suggested that the vaccination may induce heart-reactive autoantibodies, thus resulting in carditis [47].

It is important to note that while vaccine-related myocarditis has been receiving increased attention, previous case reports have suggested similar cardiovascular complications following COVID-19 infections [48]. A study by Puntmann et al*.* explored CMR outcomes in patients after COVID-19 infections and found that after two months of SARS-CoV-2 positivity, 78% of survivors had persistent cardiac involvement, with 60% showing ongoing signs of myocarditis on CMR [49]. In our study, the mean length of hospital stay was 3.9 days and 99.67% of patients studied achieved resolution of the myocarditis and/or pericarditis. These findings suggest that in contrast to infection-related myocarditis, the majority of vaccine-related myocarditis cases showed good and rapid recovery, with normalisation of ECG findings, left ventricular ejection fracture and cardiac markers often within a week [50, 51]. However, follow-up CMR was rarely performed for patients post-recovery and the long-term cardiovascular consequences of vaccine-related myocarditis remain unknown.

Nonetheless, our findings should raise the awareness of clinicians regarding the risk of developing carditis, which should be considered in individuals who present with chest pain usually around a week of vaccination, particularly within the younger male demographics. This bears increasing clinical importance as an increasing number of countries are recommending COVID-19 vaccinations for children and adolescents. Since the majority of carditis cases presented after the second dose of the COVID-19 vaccine, future research is needed regarding the safety and efficacy of additional doses in different age groups. A recent study from Israel demonstrated a 90% reduction in COVID-19-associated mortality in those who received a booster third dose of BNT162b2 at least 5 months after a second dose [52]. However, the incidence of post-vaccine carditis after an additional booster mRNA vaccination dose remains unknown and will require further investigation.

Given the global prevalence of the COVID-19 infection, in addition to the potentially long-lasting and occasionally life-threatening complications aside from carditis, it is critical to emphasize the potential benefits of the COVID-19 vaccination. Based on current evidence from this study and other published data, it largely outweighs the small, potential risk of carditis that is usually subclinical.

### Limitations

Several limitations of this study should be noted. The definitions of carditis differed between the studies, which can result in a discrepancy in the standard used for case collection. However, this is an inevitable limitation due to the difference in study design and healthcare resources across different countries. The calculation of the rate ratios was different depending on the study due to different age group stratification. Moreover, causal relationships cannot be established given the inclusion of observational studies. Additionally, it should be noted that the minimum age of vaccination varies between vaccines and countries, which can affect the interpretation of event risk amongst the younger age group. Furthermore, while the rate calculated was similar to Oster et al., the data from Krug et al*.* was derived from VAERS, which is a passive monitoring system, and thus, could be subjected to potential reporting and recall bias [14, 53, 54]. Finally, the minimum age of the paediatric patients in our included studies was 12 years old. There is still insufficient data regarding the carditis risk among patients < 12 years old and further studies are needed.

## Conclusion

Carditis is a mild and rare complication in children and adolescents after mRNA COVID-19 vaccines. The rate of carditis was significantly higher after the second dose than the first dose. Comparing the rate with COVID-19 infection, the rates of carditis after the first dose of vaccination and amongst females were lower than COVID-19 infection, but the rate of carditis after the second dose of vaccination was not different from COVID-19 infection. The carditis was often self-limiting and patients achieving full recovery within 7 days of medical treatment. Given the global prevalence of COVID-19, children should be vaccinated to protect themselves from the significant morbidity and mortality based on current evidence from this study and other published data.

## Supplementary Information

Below is the link to the electronic supplementary material.

Supplementary file1 (DOCX 17 KB)
