# Supplementary material for: COVID-19 vaccination and carditis in children and adolescents: a systematic review and meta-analysis
Source: Clin Res Cardiol. 2022 Jul 30;111(10):1161–73. doi: 10.1007/s00392-022-02070-7 (PMC9361966; doi:10.1007/s00392-022-02070-7)
Supplement: Supplementary file 1 — Supplementary file1 (DOCX 17 KB) [file 392_2022_2070_MOESM1_ESM.docx]

**Supplementary Appendix**

**Supplementary Table 1. Summary of search terms that were applied to database screening**

| **Database** | **Search terms** |
| --- | --- |
| Pubmed | ((pericarditis[Title/Abstract]) OR (myocarditis[Title/Abstract]) OR (cardiac[Title/Abstract])) |
|  | (covid19[Title/Abstract]) |
|  | (vaccin*[Title/Abstract]) |
|  | ((child*[Title/Abstract]) OR ((children*[Title/Abstract]) OR (adolescent*[Title/Abstract]) OR (paediatric [Title/Abstract]) OR (young[Title/Abstract])) |
| Embase | (pericarditis:ab,ti OR myocarditis:ab,ti OR cardiac:ab,ti) |
|  | (covid-19':ab,ti) |
|  | (vaccin*:ab,ti) |
|  | (children:ab,ti OR adolescent:ab,ti OR paediatric:ab,ti OR young:ab,ti) |
| LILACS | (miocarditis) AND (vacuna) AND (COVID) OR (coronavirus) |
|  | (pericarditis) AND (vacuna) AND (COVID) AND (coronavirus) |
| BRISA/RedTESA | (miocarditis) AND (vacuna) AND (COVID) OR (coronavirus) |
|  | (pericarditis) AND (vacuna) AND (COVID) AND (coronavirus) |
| IBECS | (miocarditis) AND (vacuna) AND (COVID) OR (coronavirus) |
|  | (pericarditis) AND (vacuna) AND (COVID) AND (coronavirus) |
| LIPECS | (miocarditis) AND (vacuna) AND (COVID) OR (coronavirus) |
|  | (pericarditis) AND (vacuna) AND (COVID) AND (coronavirus) |
| Sec. Est. Saúde SP | (miocarditis) AND (vacuna) AND (COVID) OR (coronavirus) |
|  | (pericarditis) AND (vacuna) AND (COVID) AND (coronavirus) |
| Scielo | (miocarditis) AND (vacuna) AND (COVID) OR (coronavirus) |
|  | (pericarditis) AND (vacuna) AND (COVID) AND (coronavirus) |

**Supplementary Table 2. Summary of inclusion and exclusion criteria in data screening**

| **Features** | **Inclusion criteria** | **Exclusion criteria** |
| --- | --- | --- |
| Age | ≤19 | >19* |
| Population | Human subjects | Non-human subjects |
| Type of research studies | Case reports, case studies, cohort studies | Conference papers or abstracts, reviews, systematic reviews, meta-analysis |
| Research outcome | Examines myocarditis or pericarditis in relation to the COVID-19 vaccines | Does not examine myocarditis or pericarditis as the study outcome  Does not examine in relation to COVID-19 vaccines |

**Studies that included an age range overlapping the peadiatric and adult population were included, but only the peadetric portion of the study results were included in the data*
